# Incidence of Complications Developed after the Insertion of Ventilation Tube in Children under 6 years old in 2008-2009

**Published:** 2012

**Authors:** Nader Saki, Soheila Nikakhlagh, Fariborz Salehe, Asieh Darabifard

**Affiliations:** 1 *Department of otorhinolaryngology, Imam Khomeini Hospital, Ahwaz*; 2 *General physician*

**Keywords:** Atrophy, Otitis media, Otorrhea, Tympanic membrane, Tympanosclerosis Ventilation Tube

## Abstract

**Introduction:**

One of the main treatments of chronic otitis media with effusion is ventilation of the middle ear with a ventilation tube (VT). The objective of this study was to determine the incidence and the types of VT complications in children with otitis media with effusion in Ahwaz.

**Materials and Methods:**

In this prospective study, the medical records of 208 children (52 male and 35 female) in Imam Khomeini and Apadana hospitals were reviewed. The children were between 10 months and 6 years old. The patients were followed up 12-18 months after ventilation tube insertion. We reviewed age, sex, postoperative otorrhea, eardrum atrophy, tympanosclerosis and persistent perforation. In all these patients, the indication for surgery was chronic middle ear effusion. The data were analyzed and presented as numbers and percentages using SPSS17.0.

**Results:**

Transient otorrhea occurred in 12.5% and delayed otorrhea in 8.2%. Otorrhea non-responsive to medical treatment was seen in 1.9%. Complications after tympanostomy tube extrusion included atrophy (27.8%) myringosclerosis (37.9%), and persistent perforation (2.4%).The average extrusion time was 10.5 ±5 months (ranging between 3-22 months).

**Conclusion:**

After extrusion of the ventilation tube, patients should be followed up regularly for recurrence of OME. Myringosclerosis, tympanic membrane atrophy and otorrhea are the most common complications of otitis media with effusion. However, they are generally insignificant. Consequently, in the majority of these complications, there is no need for any treatment.

## Introduction

Otitis media with effusion and eustachian tube dysfunction resulting in the effusion are among the most common diseases of the middle ear in children (1). Otitis media has a multifactorial pathogenesis, and includes such factors as viral or bacterial infections, eustachian tube dysfunction, immunologic conditions, and allergy (1, 2). Human beings have always suffered from otitis media and the consequent complications (2). Studies on Egyptian mummies of some 2600 years ago show tympanic perforation and destruction of the mastoid process (3). Evidence has also been found of middle ear diseases in the skeletons of ancient Iranians living some 900 to 1800 years ago (4).

In a book called “Diseases of the ear” in 1869, Adam Politzer presented a commendable description of otitis media and its symptoms (5). Later, BW Armstrong invented the ventilation tube, opening a new method in the treatment of the patients, an invention playing an undeniably significant role despite modern advances (6). Otitis media is currently one of the most common reasons for patients visiting doctors, clinics and medical centers, and poses high treatment costs. This disease can lead to irreparable damage if sufficient attention is not paid to diagnosis and proper treatment. The consequences include tympanic membrane atrophy, myringosclerosis, granulation tissue, eardrum perforation, cholesteatoma, behavioral changes, and sensorineural hearing loss (7, 8). The insertion of a ventilation tube (VT) in the middle ear is one of the major therapies. After VT insertion and sufficient ventilation of the middle ear, this surgical procedure can lead to consequences such as purulent otorrhea, persistent perforation of the tympanic membrane, tympanic membrane atrophy, tympanosclerosis, and relapse of effusion, which, though minor, is considered a concern (9). Most parents are willing to accept these minor risks in return for improvements in their children’s hearing impairment and reduction in ear infections (9, 10). In view of the high incidence of this disease in children, and the fact that VT is one of its major treatments, and also in view of the absence of a comprehensive study on VT complication in Ahwaz, Iran, we decided to conduct this comprehensive study on patients having undergone the drainage and VT insertion surgery in order to investigate the types of the complications and their incidence.

## Materials and Methods

In this prospective study, we studied 208 children of 10 months to 6 years of age (116 boys and 92 girls) who had undergone surgery because of chronic otitis media and VT insertion in Imam Khomeini and Apadana hospitals, Ahwaz, between September 2009 and March 2011; these children were followed up for a year and half for such complications as tympanic membrane atrophy, myringosclerosis, persistent tympanic perforation and otorrhea. Examinations were conducted using microscopes and pneumatic otoscopes, and tympanometry and audiometry were also ordered. VT insertion surgery was performed under general anesthesia. The results were analyzed using descriptive statistical methods and SPSS 17.0.

## Results

Transient otorrhea was seen in 26 children (12.5%), delayed otorrhea in 17 children (8.2%) and chronic otorrhea non-responsive to medical treatment in four children (1.9%). In one case of chronic otorrhea, a second surgery on the mastoid cavity was required, and in three cases, otorrhea was stopped upon the extrusion of the VT. The obstruction of the VT on the tympanic membrane was reported in eight cases (3.8%), granulation tissue in seven cases (3.4%), early extrusion from the membrane in 12 cases (5.7%), and displacement into the middle ear in one case (0.4%). After the extrusion of the VT, myringosclerosis was seen in 79 cases (37.9%), tympanic membrane atrophy in 58 cases (27.8%), and persistent perforation of the tympanic membrane in five cases (2.4%). Complications have been shown in Table 1 based in different age groups (Table 1).

**Table 1:** The variables in the complications of VT insertion on the tympanic membrane according to age

**Age group**	**Otorrhea**	**Persistent tympanic membrane perforation**	**Tympanic membrane atrophy**	**Myringosclerosis**
< 1 year old	8 (3.8%)	―	―	―
1-2 years old	7 (3.4%)	―	9 (4.3%)	―
2-3 years old	11 (5.3%)	2 (0.96%)	10 (4.8%)	11 (5.3%)
3-4 years old	6 (2.9%)	1 (0.48%)	10 (4.8%)	16 (7.7%)
4-5 years old	5 (2.4%)	―	11 (5.3%)	23 (11%)
5-6 years old	10 (4.8%)	2 (0.96%)	18 (8.6%)	29 (13.9%)
Total	47 (22.6%)	5 (2.4%)	58 (27.8%)	79 (37.9%)

## Discussion

In a study in 2007 in Ahwaz, Iran, conducted by Saki, et al, on 2000 grade one elementary school children, the incidence of otitis media with effusion was reported at 11.1%. In view of the high incidence, treatments and potential complications summon careful attention (5). In a study on 174 children at Shahid Sadooghi University of Medical Sciences, most VT extrusions were reported to have been performed three to six months after the surgery, and there was not a significant difference between the sexes. In this study, otorrhea was reported in 33.9% of the cases, sclerosis in 35%, persistent tympanic perforation in 3.2%, and no complications in 27.6% (8). In the present study, after the extrusion of the VT, myringosclerosis was seen in 37.9% of cases, tympanic membrane atrophy in 27.8%, and persistent perforation of the tympanic membrane in 2.4%. As can be seen in Table 1, in children under one year old, no complications were seen except otorrhea due to the short period of the study, while in older children, approaching the VT extrusion time, complications are more significant. In another, three-year, study at the Federal University of Rio de Janeiro on 75 children (aged 11 months to 10 years old) with otitis media with effusion investigating the VT complications, otorrhea was reported in 22.6%, myringosclerosis in 22.3%, and persistent perforation of the tympanic membrane in 2.1%. It was also shown in this study that VT insertion would have fewer episodes of otorrhea if accompanied by adenectomy (11). According to the results of our study, transient otorrhea was seen in 12.5% of cases, delayed otorrhea in 8.2%, and chronic otorrhea non-responsive to medical treatments in 1.9%. In another study at the ENT ward of the Hippokration Hospital, Athens, Greece, on patients with chronic otitis media having undergone VT insertion surgery, purulent otorrhea was seen in 10-26% of cases, myringosclerosis in 39-65%, segmental atrophy in 16-75%, and persistent perforation of the tympanic membrane in 3% (12). In view of the high incidence of chronic otitis media with effusion and its effect on the hearing of children at speech development age and in view of the necessity of the surgery, these complications, overall, do not have a significant effect on the individual, and such complications as tympanic membrane atrophy and myringosclerosis are more of cosmetic significance than causing serious hearing impairment. In comparison with other studies, the incidence of complications after the VT extrusion is more acceptable. 

## Conclusion

In view of the high incidence of otitis media in children, understanding such complications as tympanic membrane atrophy, myringosclerosis, and persistent perforation of the tympanic membrane are to be considered significant complications in the treatment of otitis media with effusion. Generally, these complications are insignificant and are of a cosmetic nature. Therefore, they do not require treatment.
